# The Past Winter and Present Spring

**Published:** 1842-05

**Authors:** 


### THE WESTERN JOURNAL.

Vol. V.—No. V.

LOUISVILLE, MAY 1, 1842.

### THE PAST WINTER AND PRESENT SPRING.

It may be received as a fact, that the temperature of the northern hemisphere, since last Autumn, has been higher than for many years before. On the banks of the Ohio almost every month of the winter was so mild as to excite a constant anticipation of a cold and inclement spring. March and April, however, have passed away without even their ordinary rigors; and vegetation has been unfolded a fortnight or more in advance of the usual periods. In February and March there was so much rain as to cause a great swell in the Ohio river, but the temperature still remained genial. So little ice formed below the latitude of 41°, that it was imported from the upper Mississippi, even into Cincinnati.

Notwithstanding this high and equable winter temperature, we learn that in many parts of the West a dangerous pulmonary inflammation prevailed, accompanied in some cases with a typhoid, and in others with a congestive type of fever; bringing up in the minds of the older members of the profession, recollections of the pneumonia typhoides of 1812–14. In some places this pneumonitis or bronchitis combined itself with scarlatina, constituting a very dangerous complication. In other cases a fever occurred, without the local affections characteristic of the diseases just named. Within the last two months measles have prevailed in a number of places, being in general uncommonly mild.

The object of this article is not, however, to give an account of the weather and diseases of the last few months; but to direct the attention of our brethren in the valley of the Mississippi, to the importance of making a full and permanent record of the diseases of the remarkable climatorial period through which we have just past, inasmuch as another of the same kind may not occur again for a long time. We shall be happy to receive and publish their observations.

